# Enhancing Stability of Vitamin-Fortified Protein Beverages: Optimization of Stabilizer Type and Concentration and Screening of Natural Antioxidant Combinations

**DOI:** 10.3390/foods15081392

**Published:** 2026-04-16

**Authors:** Jiaxin Li, Sumei Ru, Linru Zhu, Yingshuang Lu, Junping Wang, Yan Zhang, Lu Dong, Shuo Wang

**Affiliations:** 1 State Key Laboratory of Food Nutrition and Safety, College of Food Science and Engineering, Tianjin University of Science and Technology, Tianjin 300457, China; lijiaxintust@163.com (J.L.); sumeiru1208@163.com (S.R.); zhulinru2026@163.com (L.Z.); wangjp@tust.edu.cn (J.W.); 2 Key Laboratory of Food Quality and Health of Tianjin, Tianjin University of Science and Technology, Tianjin 300457, China; 3 Tianjin Key Laboratory of Food Science and Health, School of Medicine, Nankai University, Tianjin 300071, China; yslu@nankai.edu.cn (Y.L.); yzhang@nankai.edu.cn (Y.Z.)

**Keywords:** protein beverage, emulsion stability, microcrystalline cellulose, natural antioxidants, vitamin fortification

## Abstract

This study optimized stabilizer type and concentration, and screened natural antioxidant combinations to enhance the stability of a protein beverage fortified with vitamins A, D_2_, and D_3_. Three stabilizers—carrageenan, sodium carboxymethyl cellulose (Na-CMC), and microcrystalline cellulose (MCC)—were evaluated at 0.15–0.45% (*w*/*v*) during accelerated storage at 45 °C for 21 days. Stability was assessed using Turbiscan analysis, pH, particle size, Zeta potential, and color. MCC at 0.35% demonstrated the best stabilization, with minimal changes in Turbiscan Stability Index, particle size, and Zeta potential. Five natural antioxidants—dl-α-tocopherol, vitamin C, epigallocatechin gallate (EGCG), tea polyphenols (TP), and pyrroloquinoline quinone (PQQ)—were screened for vitamin protection using HPLC. Although vitamin C exhibited the highest in vitro DPPH radical scavenging activity (IC_50_ = 3.44 μg/mL), TP and EGCG provided superior protection of vitamins in the emulsion system. A synergistic antioxidant blend of EGCG, TP, and dl-α-tocopherol in a 4:4:2 mass ratio was identified as optimal, significantly prolonging vitamin retention over 21 days and yielding the longest predicted shelf-life (>84 days at 25 °C). These findings provide a practical formulation strategy for enhancing the physical and nutritional stability of functional protein beverages.

## 1. Introduction

The global demand for functional protein beverages has grown substantially, driven by increasing consumer awareness of health, nutrition, and convenience. Milk protein concentrates (MPCs), whey protein concentrates (WPCs), whey protein hydrolysates (WPHs), and collagen peptides are widely incorporated into beverage formulations due to their high nutritional value, bioactive properties, and technological functionality. These proteins collectively provide the eight essential amino acids required by the human body, ensuring a high-quality nutritional profile. In addition, this multi-protein combination is commonly used in commercial ready-to-drink protein beverages, where it contributes to both balanced amino acid delivery and emulsion stability [1]. Coconut oil was selected as the lipid phase due to its high content of medium-chain saturated triglycerides, which offer greater oxidative stability than the long-chain unsaturated oils typically used in beverage emulsions. Consequently, coconut oil is frequently used as the lipid component in milk protein-based beverages [2]. In parallel, fortification with fat-soluble vitamins such as A, D_2_, and D_3_ enhances the nutritional profile of these products, addressing deficiencies in specific populations and supporting bone health, immune function, and vision. Vitamins A, D_2_, and D_3_ were selected together because they are all fat-soluble, highly susceptible to oxidative degradation during storage, and frequently co-fortified in commercial protein beverages. Moreover, D_2_ and D_3_ are structural isomers, allowing direct comparison of their stability under identical conditions. Thus, these three vitamins serve as representative indicators for evaluating the broad-spectrum protective efficacy of antioxidant formulations [3]. However, the incorporation of proteins, lipids, and vitamins into a single aqueous system presents significant technological challenges, particularly regarding physical instability and chemical degradation during storage.

Protein beverages are complex colloidal systems susceptible to phase separation, creaming, flocculation, coalescence, and sedimentation, driven by gravitational forces, thermal stress, and interactions between components [4]. These instability phenomena are exacerbated during storage, especially at elevated temperatures, leading to undesirable changes in texture, appearance, and mouthfeel. Stabilizers are commonly employed to mitigate these issues by reducing interfacial tension, forming protective films around oil droplets, and increasing the viscosity of the continuous phase [5]. Common food-grade stabilizers include carrageenan, sodium carboxymethyl cellulose (Na-CMC), and microcrystalline cellulose (MCC), each exhibiting distinct mechanisms of stabilization. Carrageenan forms gel networks through helical aggregation, Na-CMC increases viscosity via polymer entanglement, and MCC acts as a Pickering-type stabilizer through insoluble particle adsorption at interfaces [6]. Despite their widespread use, comparative studies on their long-term efficacy in high-protein, vitamin-fortified beverages under accelerated storage conditions remain limited. Existing studies have compared stabilizers such as carrageenan, CMC, and pectin in fruit juice-milk systems or acidified whey protein beverages, and have evaluated the effects of different stabilizer combinations on protein beverage stability [7]. However, these studies did not (i) evaluate their performance under identical accelerated conditions (45 °C) using a unified comparative framework or (ii) provide a direct comparative ranking of their individual stabilizing efficacy. The present study addresses these gaps by systematically evaluating the long-term efficacy of MCC, Na-CMC, and carrageenan as single stabilizers in a high-protein beverage under accelerated storage at 45 °C, using physical stability indicators (Turbiscan analysis, pH, particle size, Zeta potential, and color) as the primary assessment criteria.

Beyond physical instability, oxidative deterioration poses a critical threat to the quality and nutritional value of protein beverages. Lipids, even at low concentrations, are susceptible to autoxidation, generating hydroperoxides, free fatty acids, and secondary oxidation products that compromise flavour, colour, and vitamin stability [8]. Fat-soluble vitamins, particularly vitamins A and D, are highly labile and degrade rapidly under oxidative stress, light, and elevated temperatures, diminishing the product’s nutritional efficacy [9]. To counteract oxidation, synthetic antioxidants such as butylated hydroxyanisole (BHA) and butylated hydroxytoluene (BHT) have been traditionally used, but growing consumer preference for clean-label products has shifted attention toward natural antioxidants. Natural antioxidants, including epigallocatechin gallate (EGCG), tea polyphenols (TP), vitamin C, pyrroloquinoline quinone (PQQ) and dl-α-tocopherol, exhibit potent radical-scavenging activities and are increasingly explored as functional additives [10]. However, their effectiveness in complex emulsion systems is governed not only by intrinsic antioxidant capacity but also by factors such as polarity, partitioning behaviour, molecular stability, and interactions with other matrix components [11]. In particular, the synergistic effects between hydrophilic and lipophilic antioxidants, which enable phase-distributed protection and redox regeneration, are of great interest. Hydrophilic antioxidants (e.g., vitamin C, EGCG, and TP) localize at the oil-water interface and in the aqueous phase, where they scavenge free radicals generated in these compartments. Lipophilic antioxidants (e.g., dl-α-tocopherol) partition into the oil phase, protecting lipids and lipophilic vitamins from oxidation within the droplet interior. This spatial complementarity ensures comprehensive coverage across all phases of the emulsion. Furthermore, certain hydrophilic antioxidants, particularly vitamin C, are known to regenerate oxidized tocopherols at the interface, creating an antioxidant network that sustains protection over extended periods [12]. However, they remain poorly characterized in protein-based beverages containing multiple labile vitamins.

Furthermore, while in vitro antioxidant assays such as DPPH radical scavenging provide preliminary screening data, they often fail to predict performance in real food systems due to differences in microenvironment, partitioning, and interactions with proteins and stabilizers [13]. Therefore, a systematic approach combining physical stability assessment, chemical analysis of vitamin retention, and shelf-life modeling is essential to develop effective formulations. Accelerated shelf-life testing using the Q_10_ model offers a practical means to estimate product stability under normal storage conditions based on degradation kinetics at elevated temperatures [14].

Given these considerations, the present study was undertaken with the following objectives: (1) to screen three stabilizers—carrageenan, Na-CMC, and MCC—for their ability to maintain the physical stability of a model protein beverage during accelerated storage; (2) to optimize the concentration of the most effective stabilizer; (3) to evaluate five natural antioxidants for their capacity to protect vitamins A, D_2_, and D_3_ during storage; and (4) to identify an optimal synergistic antioxidant blend that maximizes vitamin retention and extends shelf-life. By integrating physicochemical characterization, chromatographic vitamin analysis, and shelf-life prediction, this research aims to provide a comprehensive formulation strategy for developing high-quality, nutritionally stable protein beverages suitable for commercial application.

## 2. Materials and Methods

### 2.1. Materials

MPC, WPC, WPH, collagen peptides, coconut oil, vitamin A, vitamin D2, and vitamin D3 were procured from Sigma Chemical Co., Sydney, Australia. All other chemicals and reagents were of AR or HPLC grade. C_18_ solid-phase extraction (SPE) columns (500 mg) were purchased from Agela (Sydney, Australia). Food-grade antioxidants and stabilizers were supplied by Shanghai Yuanye Bio-Technology Co., Ltd. HPLC-grade reagents were purchased from Spectrum Chemical Mfg. Corp. (Gardner, CA, USA). All other analytical-grade reagents were obtained from Kemiou Chemical Reagent Co., Tianjin, China.

### 2.2. Preparation of Protein Beverage Samples

The beverage mixture comprised 7% (*w*/*v*) MPC, 1.5% (*w*/*v*) WPC, 0.5% (*w*/*v*) WPH, 1% (*w*/*v*) collagen peptides, 0.25% (*w*/*v*) coconut oil, 45 μg/100 mL of vitamin A, 2 μg/100 mL of vitamin D2 and D3, and potable water. Solid particles were dispersed via high-speed homogenisation at 1300 rpm for 20 min. Subsequently, the mixture was portioned into 300 mL opaque, food-grade plastic bottles with lids. The sealed bottles were heated in a 60 °C water bath for 30 min. After treatment, aliquots were aseptically collected for subsequent use. The samples were placed in a 45 °C incubator for 21 days. Weekly sampling was conducted to measure various indicators, with each sampling procedure performed under sterile conditions. All sample preparation and handling were performed under subdued light. Samples were stored in opaque bottles in a dark incubator. Sampling was performed weekly. Sampling tools were autoclaved, and all operations were performed in a laminar flow cabinet.

### 2.3. Screening of Stabilizers and Natural Antioxidants

#### 2.3.1. Stabilizer Screening: Type and Concentration

In accordance with Chinese Standard GB 2760-2014, 0.1% (*w*/*v*) carrageenan, MCC, and Na-CMC, respectively, were incorporated into the base formulation described in Section 2.2. Through a comprehensive evaluation of sample physical stability during storage, the most effective stabilizer type was selected for subsequent experiments. The selected optimal stabilizer was then incorporated into the formulation at four concentration gradients: 0.15%, 0.25%, 0.35%, and 0.45% (*w*/*v*). Employing the same evaluation methodology as the stabilizer selection, the optimal addition concentration was determined. The resulting optimal stabilizer system served as the fixed matrix for subsequent antioxidant screening.

#### 2.3.2. Natural Antioxidants Screening: Types and Formulations

Add 10 mg/100 mL of dl-α-Toc, vitamin C, EGCG, TP, or PQQ, respectively, to the aforementioned optimized stabilizer systems. dl-α-Tocopherol, being lipid-soluble, was first dissolved in a portion of the coconut oil to facilitate its dispersion within the lipid phase. Vitamin C, EGCG, and TP are water-soluble and were directly dissolved in the aqueous protein solution. PQQ can also be dissolved in solution at a concentration of 10 mg/100 mL. Samples without added antioxidants served as the blank control group. The superior protective effects were screened and identified using the DPPH radical scavenging assay and vitamin retention analysis. Based on the screening results by type, several single antioxidants with the best effects were selected for compounding experiments at different ratios to identify the combination and proportion with the optimal synergistic effect.

### 2.4. Characterization and Analytical Methods

#### 2.4.1. Delta Backscattering Profiles and Turbiscan Stability Index Value

The delta backscattering profiles and Turbiscan stability index value were analyzed using Turbiscan Lab^®^ Expert (Formulaction S.A., Toulouse, France) at 60 °C for 1 h. The change in ∆BS signal was calculated as the difference between backscatter signals from 0 to 60 min, with ∆BS plotted on the y-axis and sample height (h, mm) on the x-axis. TSI values were calculated based on backscatter data, incorporating the thickness of formed deposits and flocculation. TSI values are represented as TSI-GLOBAL (TSIG), TSI-Bottom (TSIB), TSI-Medium (TSIM), and TSI-TOP (TSIT). Higher TSI values indicate lower sample stability during the Turbiscan scanning process [15].

#### 2.4.2. pH Value Measurement

For each sample, 10 mL was taken, thoroughly homogenized, and then the pH was measured and recorded on day 0 and day 21 using a calibrated ChemTron Lab 845 (JULABO GmbH, Seelbach, Germany) pH meter. No pH adjustment was performed during sample preparation, as the formulations were evaluated under their native pH conditions.

#### 2.4.3. Particle Size and Zeta Potential Analysis

Measure the particle size and Zeta potential of the sample using a Nanoparticle Particle Size Analyser and Zeta Potential Analyser (Bettersize Instruments Ltd., Dandong, China). For Zeta potential measurements, dilute the sample to 0.1% (*v*/*v*) using Millipore water and conduct measurements at 25 °C. For particle size analysis, dilute the sample 100-fold with Millipore water [16].

#### 2.4.4. Analysis of Colour

The samples’ colour analysis was measured using a KONICA MINOLTA SPECTRO PHOTO METER CM-5 Chroma Meter (KONICA MINOLTA, INC., Tokyo, Japan). Determine the colour difference (ΔE) variation in the sample. Measure the L* value, a* value, and b* value. Calculations shall be performed using the following equation:

(1) $$\Delta E=\sqrt{{\left(L_{1}^{*}-L_{2}^{*}\right)}^{2}+{\left(a_{1}^{*}-a_{2}^{*}\right)}^{2}+{\left(b_{1}^{*}-b_{2}^{*}\right)}^{2}}$$

#### 2.4.5. Evaluation of Antioxidant Properties

The DPPH radical scavenging assay was employed to determine antioxidant activity, with the concentration yielding a 50% inhibition (IC_50_) value used to assess the reducing capacity of each antioxidant. Subsequently, absorbance was measured at 517 nm using a Shimadzu UV-2700 spectrophotometer (Shimadzu Co., Kyoto, Japan). A blank solution underwent similar treatment to obtain the absorbance for the control group. DPPH (0.1 mM in ethanol) was mixed with sample (50 μL) and 2.95 mL DPPH solution, incubated at 25 °C for 30 min in the dark, and measured at 517 nm. DPPH radical scavenging activity was calculated according to Equation (2), where Ac denotes the absorbance of the control group, and A_0_ represents the absorbance of the sample.

(2) $$Inhibition\;(\%)=(\frac{Ac-A_{0}}{Ac})\times 100$$

#### 2.4.6. Determination of Vitamins A and D2/D3 by Solid Phase Extraction-HPLC

Sample Preparation. Accurately weigh 10 g of sample, and add 12 mL of 2% (*w*/*v*) BHT ethanol solution, and 6 mL of 10% (*w*/*v*) potassium hydroxide solution. Mix thoroughly, fill with nitrogen gas, and perform the saponification reaction at 50 °C in a water bath with shaking for 30 min. Add 20 mL isopropanol/hexane (1:1) to the saponified solution. Vortex briefly, then centrifuge at 6000 rpm for 3 min. Transfer the upper clear layer, add 25 mL of water, shake thoroughly, and centrifuge again for 3 min. Collect the upper organic phase for subsequent use. Pass the reserved solution through a column, elute with 6 mL ethyl acetate/hexane solution (5:95), and then elute with 6 mL ethyl acetate/hexane solution (15:85). Evaporate the eluate to dryness under nitrogen at 30 °C. Add 2 mL methanol, vortex, and filter through a 0.22 μm organic-compatible membrane for instrumental analysis. All operations were conducted in the absence of light.

Analysis of vitamins A, D_2_, and D_3_. A Phenomenex C18 column (4.6 mm × 250 mm, particle size 5 µm) was used with the following parameters: flow rate: 0.8 mL/min; injection volume: 20 µL; column temperature: 30 °C; detection wavelength: vitamin A: UV 325 nm, vitamin D_2_ and vitamin D_3_: UV 264 nm. The HPLC analysis was performed using gradient elution. The mobile phase consisted of water (A) and methanol (B). The gradient program was as follows: 0–13 min, 90% B; 13–15 min, B increased from 90% to 100%; 15–26 min, 100% B; 26–30 min, B decreased from 100% to 90%. The total run time was 30 min. The relationship between peak area and concentration was described using linear regression equations. Corresponding equations were established using calibration curves with vitamin A concentrations of 0.5, 2.0, 3.0, 4.0, and 5.0 μg/mL, and vitamin D_2_ and vitamin D_3_ concentrations of 0.1, 0.2, 0.4, 0.6, and 0.8 μg/mL.

### 2.5. Shelf-Life Prediction

The shelf-life end-point was defined according to GB 28050-2011, which specifies that vitamin A and vitamin D content must remain within 80–180% of the labelled value throughout the product shelf-life. The Q_10_ model was selected following the Chinese National Standard T/CNFIA 001-2017 “General Guide for Food Shelf Life Determination”, which recommends the Q_10_ method for accelerated shelf-life testing of foods. The shelf life of dairy beverages stored at 45 °C was predicted using the Q10 model. Q10 was selected as 2 and calculated using the following equation, where θ*_s_*(*T*) denotes the shelf life at 25 °C. According to this standard, a default Q_10_ value of 2 is adopted for most foods, based on the general principle that the rate of chemical degradation doubles for every 10 °C increase in temperature. θ(*T*’) represents the shelf life obtained from accelerated destructive testing at 45 °C, and Δ*T_a_* is the temperature difference between the higher temperature (*T*’) and the actual storage temperature (*T*), expressed in degrees Celsius (°C).

(3) $$\theta_{s}\left(T\right)=\theta_{s}\left(T^{\prime }\right)\times Q_{10}^{\Delta T_{a}/10}$$

### 2.6. Statistical Analysis

All experiments were repeated three times. Data are presented as mean ± standard deviation (STD). Statistical analysis was performed using Tukey’s honest significant difference (HSD) post hoc test with SPSS 31.0.2 (IBM Corporation, Armonk, NY, USA). *p* values ≤ 0.05 were considered statistically significant.

## 3. Results and Discussion

### 3.1. Screening of Stabilizer Type

#### 3.1.1. Delta Backscattering Profiles and Turbiscan Stability Index Value of Different Stabilizer Types

The Turbiscan Lab analyser was employed to investigate the stability of the system during storage, revealing emulsions, coagulation, flocculation, sedimentation, and other instability phenomena [17]. A systematic analysis of the destabilisation process in milk protein beverages with different stabilizer-stabilised systems during accelerated storage was conducted via backscatter (ΔBS) mapping, illustrating the relationship between backscattered light intensity, height, and time. Samples exhibiting high positive BS values typically indicate flocculation or sedimentation, whereas negative BS values denote the emergence of clearer fractions. Minimal changes over time indicate high stability, whereas significant variations signify system instability. The results demonstrated irreversible phase separation in all beverage systems. In the Na-CMC system, bottom ΔBS steadily increased (8% to 17%), the middle section (15–30 mm) showed a slight decrease of 3%, and the top section (>30 mm) also exhibited an upward trend (10% to 15%). This indicates that samples with Na-CMC addition developed sedimentation at the bottom, slight clarification in the middle, and a distinct emulsion stratification at the top during storage. In the carrageenan system, the bottom ΔBS exhibited an upward trend (7% to 15%), while the middle section showed a sharp decrease (30%). This indicates that the sample exhibited slight precipitation at the bottom during the initial storage period. However, as time progressed, the middle and upper sections became clearer, with significant stratification occurring. This suggests poor system stability. This may be attributed to the fact that although carrageenan possesses excellent gelling, thickening, and emulsion-stabilising properties, owing to its rapid gelation properties, initial stability is favourable, yet stratification and quality deterioration accelerate during prolonged storage. Similar findings have been reported in prior literature [18]. The MCC system exhibited minimal overall change: an increase in the bottom layer (5% to 9%) and a negative change in the middle layer ΔBS (2%). This indicates the slowest accumulation of sedimentation in this system, effectively inhibiting flocculation and stratification, and demonstrating optimal stability (Turbiscan scanning spectra are shown in the Supplementary File Figures S1–S3).

**Figure 1:**
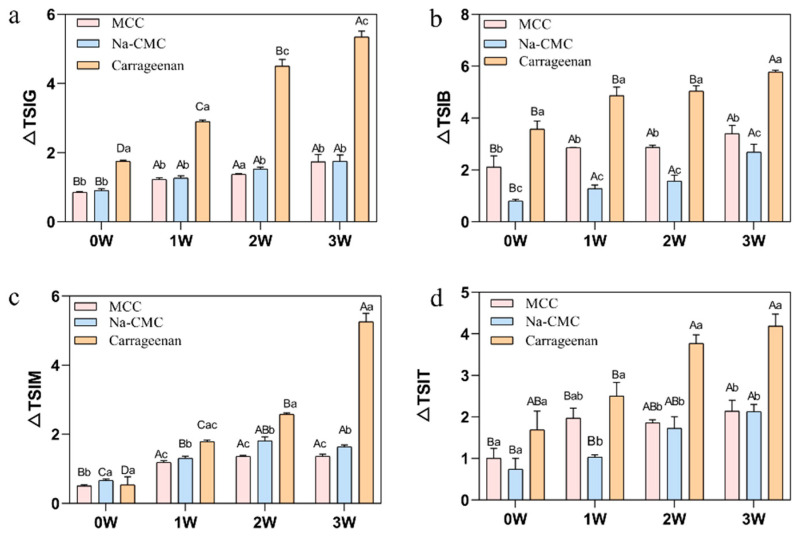
Trends in TSI values over 1–3 weeks of storage with addition of MCC, Carrageenan, and Na-CMC to the system ((**a**) TSIG overall, (**b**) TSIB bottom, (**c**) TSIM middle, (**d**) TSIT top). Different uppercase letters indicate significant differences between samples of the same type at different sampling times; different lowercase letters indicate significant differences between samples at the same sampling time (*p* < 0.05).

Concurrently, the TSI values for all samples during storage were determined (Figure 1). An increase in TSI values indicates reduced sample stability, manifested through clarification, emulsification, flocculation, or precipitation. Compared to week 0, the total TSI values increased by 39.37–189.89% (Na-CMC), 35.29–174.56% (MCC), and 36.14–920.03% (Carrageenan). The exceptionally wide range for carrageenan (36.14% to 920.03%) indicates that this stabiliser performed relatively well during the initial storage period but underwent catastrophic destabilisation in the later stages, with a nearly 10-fold increase in TSI by week 3. In contrast, MCC showed the narrowest range (35.29–174.56%) and the lowest absolute TSI values throughout storage, indicating more consistent and superior long-term stability. Na-CMC exhibited intermediate behaviour, with a moderate TSI increase range (39.37–189.89%). As storage time extended, the stability of each stabilizer system decreased significantly, with the trend being: MCC < Na-CMC < Carrageenan.

Overall, MCC demonstrated superior performance in delaying emulsion separation and instability, whereas Carrageenan exhibited the most pronounced decline in stability during prolonged storage.

#### 3.1.2. pH Value Variation of Different Stabilizer Types

**Figure 2:**
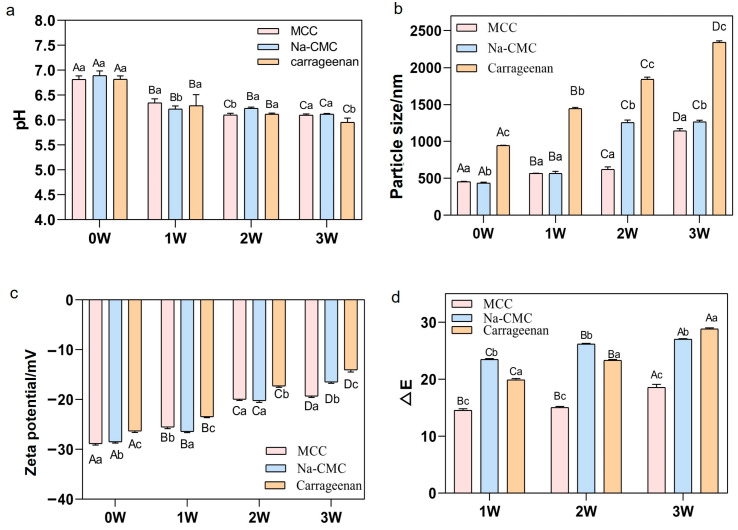
Changes in (**a**) pH, (**b**) particle size (nm), (**c**) Zeta potential (mV), and (**d**) ΔE during storage with the addition of MCC, Na-CMC, and carrageenan to the system. Different uppercase letters indicate significant differences between samples of the same type at different sampling times; different lowercase letters indicate significant differences between samples at the same sampling time (*p* < 0.05).

The pH value of the samples gradually decreased during storage, consistent with previous research findings indicating a pH decline when storage temperatures are elevated during emulsion preservation [19]. This is attributable to oxidative reactions occurring in fats during storage, yielding products such as free fatty acids and small-molecule organic acids, thereby causing the pH decline. The results indicate that the magnitude of pH decrease was MCC sample < Na-CMC sample < Carrageenan sample, further demonstrating MCC’s superiority in inhibiting emulsion oxidation (Figure 2a).

#### 3.1.3. Particle Size and Zeta Potential Analysis of Different Stabilizer Types

It has been reported that adding stabilizers can reduce the average particle size and polydispersity index, thereby improving the shelf stability of beverages [20]. Figure 2b shows the effects of carrageenan, Na-CMC, and MCC on beverage particle size. The particle size of the samples gradually increased with storage time, indicating a significant decline in system stability over time. This may result from particle coalescence due to protein denaturation and aggregation during storage, leading to increased particle size. Specifically, Carrageenan samples exhibited significant particle size increase by the first week of storage; Na-CMC samples showed marked changes by the second week; and MCC samples experienced dramatic particle size changes by the third week. Results indicate that MCC more effectively delays particle size growth, thereby better maintaining emulsion stability.

As storage time extended, the absolute values of Zeta potential gradually decreased across all samples, indicating a progressive decline in system stability (Figure 2c). This may stem from protein denaturation and aggregation during storage, reducing the specific surface area and consequently diminishing the available negative charge on particle surfaces, thereby lowering absolute Zeta potential values [21]. The trend revealed that samples supplemented with MCC exhibited the least change, followed by Na-CMC, while carrageenan samples demonstrated the most pronounced Zeta potential variation. These results indicate that MCC provides the most effective maintenance of the system’s storage stability.

#### 3.1.4. Colour Evaluation of Different Stabilizer Types

Colour serves as a fundamental indicator of food quality and is crucial for consumer acceptance. As storage duration increased, the ΔE value progressively rose, indicating significant colour changes in the emulsion system during high-temperature storage. This may result from Maillard reactions and other chemical processes altering the emulsion’s colour (Figure 2d). Protein content, heating process, and heating temperature all significantly influenced the L* value, consistent with previous research [22]. The trend in the ΔE was MCC samples < Na-CMC samples < Carrageenan samples, indicating that MCC can, to some extent, inhibit colour deterioration in the emulsion during storage.

In summary, the results from the Stability Analysis, pH, potential, particle size, and colour evaluation indicate that the emulsions prepared using MCC exhibit superior long-term stability.

### 3.2. Screening for the Optimal Addition Rate of Stabilizers

#### 3.2.1. Delta Backscattering Profiles and Turbiscan Stability Index Value of Different Stabilizer Concentrations

The destabilisation process of protein beverages with varying microcrystalline cellulose additions during accelerated storage was systematically analysed via backscatter (ΔBS) mapping (Turbiscan scanning spectra are shown in the Supplementary File Figures S4–S7).

For the unstable concentrations (0.15% and 0.25% MCC), both exhibited rapid destabilisation. The 0.15% MCC system exhibited a 17% increase in top ΔBS and a sharp decline in the middle-upper layer during the initial storage phase, with this trend intensifying to 50% in the later stages. This indicates rapid emulsion separation at the top, extensive clarification in the middle-upper layer, sedimentation at the bottom, and pronounced stratification. The 0.25% MCC system exhibited a substantial 47% decrease in the middle-upper layer during the later storage period. Although this decline was less pronounced than in the 0.15% sample, significant clarification and emulsion stratification in the middle-upper layer persisted, indicating generally moderate overall stability. In contrast, the 0.35% MCC system exhibited the smallest and most stable increase in the bottom layer throughout storage (7% to 11%). Although the top layer decreased by 33% in the later stage, the trend remained relatively stable. This indicates that this concentration effectively delays sediment formation, significantly mitigates the extent and rate of middle-upper layer clarification and stratification, and demonstrates the best overall stability. For the excess concentration (0.45% MCC), the 0.45% MCC system exhibited minimal variation in the bottom layer. However, the middle and upper layers experienced a 53% reduction in ΔBS, with layering becoming more pronounced compared to the 0.35% system.

**Figure 3:**
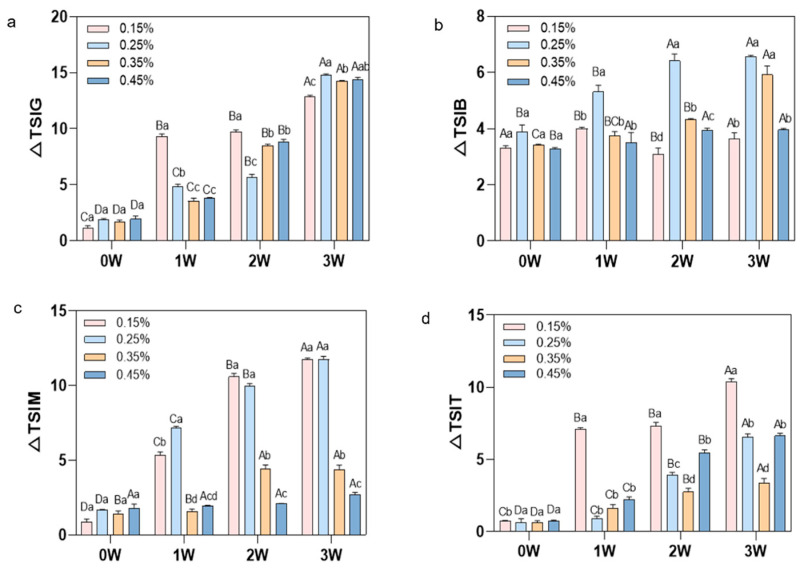
Changes in TSI values during storage periods of 1–3 weeks with the addition of 0.15%, 0.25%, 0.35%, and 0.45% MCC to the system ((**a**) TSIG overall, (**b**) TSIB bottom, (**c**) TSIM middle, (**d**) TSIT top). Different uppercase letters indicate significant differences between samples of the same type at different sampling times; different lowercase letters indicate significant differences between samples at the same sampling time (*p* < 0.05).

The TSI values of emulsion systems with different MCC concentrations over 1, 2, and 3 weeks at 45 °C storage exhibited a trend of 0.35% < 0.45% < 0.25% < 0.15%. Statistical analysis revealed that at Week 0, no significant differences were detected among any concentrations. At Weeks 1 and 2, 0.15% and 0.25% showed significantly higher TSI than 0.35% and 0.45% (*p* < 0.05), while 0.35% vs. 0.45% showed no significant difference. At Week 3, a significant difference emerged between 0.35% and 0.45% (*p* < 0.05). The sample containing 0.35% MCC exhibited optimal stability throughout the storage period, demonstrating the lowest TSI value across all concentration gradients (Figure 3). This indicates its relatively effective suppression of emulsion separation, sedimentation, and instability phenomena.

It has been reported that excessive additives weaken gel strength [23]. Research indicates that limited protein networks can only stabilize a finite quantity of emulsion; over-addition causes leakage and disrupts protein cross-linking. Consistent with the findings of Wang et al. [24] in oat beverage systems, the stability of MCC-stabilised emulsions does not increase monotonically with concentration. Within this system, the interfacial film formed by components such as whey protein also possesses a limited capacity to support MCC particles. When MCC is added at moderate levels (e.g., 0.35%), it synergises with interfacial proteins to enhance interfacial strength. However, excessive addition (e.g., 0.45%) may cause surplus MCC to compete with proteins for oil-water interface positions or form bulk aggregates that disrupt the continuity of the interfacial film. This increases susceptibility to coalescence and flotation, manifesting as accelerated increases in TSI values.

The three-dimensional network formed by MCC acts as a suspension agent, reducing particle aggregation during sterilisation and storage. However, excessively high concentrations may paradoxically promote emulsion aggregation and phase separation. Previous research has confirmed this phenomenon [6]. In summary, 0.35% represents the optimal addition concentration, most effectively inhibiting precipitation and stratification throughout the storage period while maintaining a uniformly stable system.

#### 3.2.2. pH Value Variation of Different Stabilizer Concentrations

**Figure 4:**
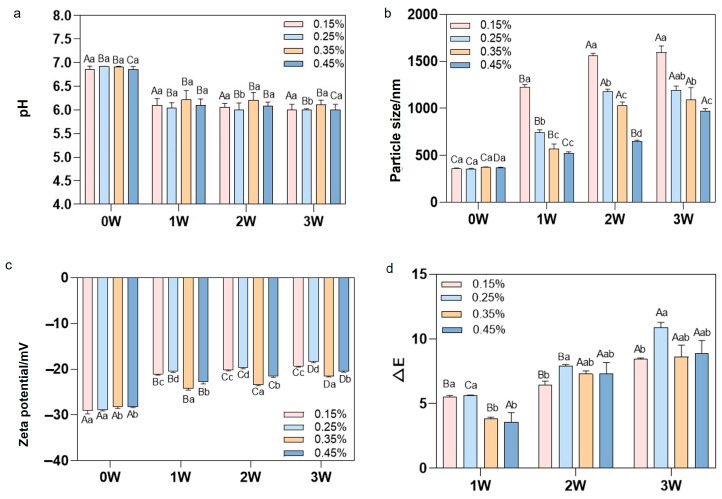
Changes in (**a**) pH, (**b**) particle size (nm), (**c**) Zeta potential (mV), and (**d**) ΔE during storage with the addition of 0.15%, 0.25%, 0.35%, and 0.45% MCC, respectively. Different uppercase letters indicate significant differences between samples of the same type at different sampling times; different lowercase letters indicate significant differences between samples at the same sampling time (*p* < 0.05).

The pH value gradually decreased throughout storage. Samples supplemented with 0.35% MCC exhibited the smallest pH variation, indicating superior inhibition of reactions such as fat oxidation, free fatty acid formation, and small-molecule organic acid production compared to other concentrations (Figure 4a). Research indicates that stabilizers at appropriate concentrations can maintain relatively stable pH levels in emulsions throughout their shelf life. Conversely, high stabilizer concentrations may induce significant pH shifts, primarily due to competitive interactions between stabilizers and proteins at the interface [25]. When the stabilizer concentration exceeds a critical threshold, it partially displaces proteins adsorbed at the interface. This exposes more ionisable amino acid residues (e.g., carboxyl or amino groups), altering their proton-binding/releasing behaviors. Concurrently, the self-ionization of ionic stabilizers or the micellar environment they form directly disrupts the bulk phase’s ionic equilibrium and proton distribution. This phenomenon of coupled changes in interfacial composition and protein conformation renders pH a sensitive indicator of alterations within the physicochemical microenvironment of emulsion systems. It further suggests that, in practical applications, stabilizer concentrations should be controlled within ranges that avoid such pronounced perturbations to proton equilibrium.

#### 3.2.3. Particle Size and Zeta Potential Analysis of Different Stabilizer Concentrations

Figure 4b,c demonstrate that particle size progressively increased with storage time across all concentration samples, indicating the gradual manifestation of system instability due to protein aggregation (with 0.15% being the least stable). The particle size change was minimal for the 0.35% MCC concentration, demonstrating superior inhibition of protein coagulation and particle enlargement. The absolute Zeta potential values showed a decreasing trend during storage, reflecting reduced electrostatic repulsion and further confirming the destabilisation of the emulsion systems. This aligns with the pH trend observed across samples containing varying MCC concentrations. It has been reported that at lower pH values, electrostatic repulsion between particles diminishes, leading to emulsion aggregation. This increase and decrease in electrostatic repulsion also explain why the average emulsion particle size increases as pH decreases. A reduction in whey protein solubility observed near the isoelectric point may also contribute to droplet flocculation, as investigated by Rao et al. [26].

#### 3.2.4. Colour Evaluation of Different Stabilizer Concentrations

Figure 4d demonstrates that as storage time increases, the change in ΔBS initially grows larger with rising MCC concentration before diminishing. Samples incorporating 0.35% MCC exhibited the smallest variation in values. This may be attributed to excess MCC inducing flocculation within the emulsion, thereby enhancing light scattering and rendering the emulsion uneven with a greyish cast. In summary, a 0.35% MCC concentration effectively mitigates colour deterioration during storage.

Overall, the 0.35% MCC addition concentration demonstrated optimal performance in both system stability and quality preservation.

### 3.3. Screening of Natural Antioxidant Types

#### 3.3.1. Evaluation of Antioxidant Potential

**Figure 5:**
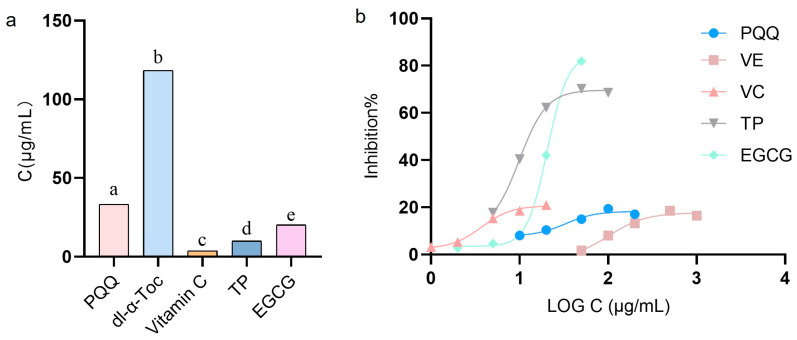
IC_50_ (in μg/mL) values of PQQ, dl-α-Toc, vitamin C, TP, and EGCG (**a**); plot of radical scavenging percentage at different concentrations of PQQ, dl-α-tocopherol, vitamin C, TP, and EGCG (**b**). Different lowercase letters denote significant differences at *p* < 0.05.

The reducing capacity of antioxidants was determined using the concentration yielding a 50% inhibition (IC_50_) value (Figure 5a). The average percentage of DPPH radical scavenging activity at different antioxidant concentrations is shown in Figure 5b. IC_50_ (in μg/mL) values: vitamin C (3.44 ± 0.32) < TP (10.05 ± 0.07) < EGCG (20.15 ± 0.34) < PQQ (33.33 ± 0.23) < dl-α-Toc (119.47 ± 0.31). To determine antioxidant activity in vitro, vitamin C > TP > EGCG > PQQ > dl-α-Toc (*p* < 0.05). The significance of determining the IC_50_ value lies in its ability to quantitatively compare the strengths of free radical scavenging capacities of different antioxidants in vitro, while also providing a direct reference for the appropriate addition concentrations of various antioxidants in subsequent emulsion systems [11]. The lower DPPH radical scavenging activity of dl-α-tocopherol observed in this study can be attributed to the inherent characteristics of the DPPH assay. This assay is conducted in polar organic solvents, whereas dl-α-tocopherol is lipophilic; its reaction kinetics with DPPH are known to be strongly solvent-dependent. Moreover, comparative studies have consistently shown that various polyphenols exhibit stronger DPPH scavenging effects than dl-α-tocopherol, reflecting the assay’s inherent bias toward polar and amphiphilic antioxidants. Thus, the lower DPPH activity of dl-α-tocopherol does not indicate inferior intrinsic antioxidant capacity, but rather highlights the importance of using complementary assays for evaluating lipophilic antioxidants. It remains unclear whether the antioxidant effects observed in vitro can be replicated in actual emulsion systems. Therefore, further studies in emulsion systems are needed to verify this.

#### 3.3.2. Vitamin Analysis by HPLC

The study assessed the protective efficacy of individual antioxidants on fat-soluble vitamins during accelerated storage. A quantitative method was established. The chromatograms of reference standards and methodological evaluation data are provided in the Supplementary File (Figure S8 and Table S1), and the experimental results demonstrate that this method is highly stable and suitable for detecting vitamins. The higher vitamin A content in the EGCG group at week 0 is attributed to EGCG’s protective effect during the 60 °C heat treatment prior to storage. EGCG’s multiple phenolic hydroxyl groups enable it to chelate metal ions and scavenge thermally generated free radicals, while its ability to form complexes with whey proteins localises it at the oil-water interface, protecting fat-soluble vitamins during processing. This early protection contributes to the higher initial vitamin A concentration and subsequent sustained retention in EGCG-containing formulations [11]. On day 7 of storage, vitamin A retention was highest in the vitamin C and TP groups, while vitamin D_3_ retention was optimal in the EGCG and TP groups. No significant differences in vitamin D_2_ content were observed across groups. In contrast, in the control group (without any antioxidant), all three vitamins fell below the limit of detection as early as day 7 (Table 1). Notably, water-soluble vitamin C provides better protective effects against lipophilic vitamins in the early stages than other antioxidants. This may be attributed to vitamin C’s ability to position itself at the oil-water interface and regenerate other antioxidants. Therefore, vitamin C offers better immediate protection against early oxidation. In contrast, dl-α-tocopherol is highly lipophilic and remains partitioned within the oil phase, limiting its access to pro-oxidants in the aqueous phase during the initial storage period. However, by day 14, vitamin C’s protective effect weakens due to thermal decomposition, while TP and EGCG maintain superior activity. This indicates that the long-term stability of the antioxidant itself is a key determining factor for sustained vitamin protection [27]. For the PQQ group, it is extremely sensitive to heat and may degrade under accelerated storage conditions. Furthermore, only these two groups yielded detectable levels of vitamin D_2_ and D_3_, whereas target vitamin concentrations in the remaining groups (particularly dl-α-tocopherol and PQQ) fell below the limit of detection (Table 1, and comparative HPLC analysis of samples is shown in the Supplementary File Figure S9). This disparity correlates closely with the properties of each antioxidant: vitamin C, dl-α-Tocopherol, and PQQ readily undergo thermal degradation, oxidation, or structural ring-opening under storage conditions [28], whereas TP and EGCG exhibit higher molecular stability [29], sustaining their radical scavenging capacity. Overall, the retention efficacy of individual protectants for vitamins ranked as follows: TP > EGCG > vitamin C > PQQ > dl-α-Toc. TP demonstrated comprehensive and significant protective advantages, attributable to its stable chemical structure and sustained antioxidant activity, enabling it to effectively maintain the stability of all three sensitive vitamins throughout the 14-day storage period.

### 3.4. Optimisation of the Addition Ratio for Blended Natural Antioxidants

Based on the optimal protective efficacy of EGCG and TP on fat-soluble vitamins in the system, these antioxidants were selected for compounding experiments at different ratios to identify the combination with optimal synergistic effect. Previous literature [30] indicates that synergistic interactions between antioxidants are influenced by concentrations and varying systems. Guzman et al. [31] described that composite antioxidants comprising TBHQ+BHA, TBHQ+PG, and TBHQ+resorcinol exhibited varying antioxidant efficacy in distilled poultry fat and soybean oil depending on their mixing ratios. Based on the findings in Section 3.3.2, establishing a composite antioxidant system in this framework utilising dl-α-Toc (lipophilic) alongside TP and EGCG (hydrophilic) operates on the principle of achieving phase partitioning complementarity and redox synergy. dl-α-Toc primarily distributes within the lipid phase of emulsions and at the interfacial membrane, enabling targeted protection of fat-soluble vitamins and termination of lipid peroxidation chain reactions. Its synergistic interactions with other antioxidants are well-documented in the literature [32]. Meanwhile, TP and EGCG exert radical scavenging effects in the aqueous phase and can regenerate dl-α-Toc through the reduction of its free radicals, thereby more effectively maintaining the stability of multiple fat-soluble nutrients during storage. However, the precise and optimal addition ratio remains uncertain. Consequently, different addition levels of the mixture (EGCG+ TP+ dl-α-Toc) (Table 2) were established, and the vitamin retention rate of the samples was measured to determine the optimal addition ratio.

Results indicate that the higher week 0 vitamin levels in the 4:4:2 group compared to other ratio groups (Table 2) reflect the protective effect of the balanced antioxidant mixture during the 60 °C heat treatment step prior to storage. The 4:4:2 ratio likely achieves optimal phase distribution, with hydrophilic EGCG and TP protecting the oil-water interface and lipophilic dl-α-tocopherol protecting the oil phase. This coordinated protection minimises vitamin degradation during processing, resulting in higher baseline concentrations. Suboptimal ratios (e.g., 2:6:2, 5:3:2, 6:2) fail to achieve this balance, leading to greater vitamin loss during heat treatment and the initial storage period [33]. After 7 days of storage, the 4:4:2 and 3:5:2 groups showed significantly greater protective effects for all three vitamins than the other groups (*p* < 0.05). By day 14, the 4:4:2 group exhibited the highest retention rates for both vitamin D_2_ and vitamin D_3_. At the storage endpoint (day 21), the 4:4:2 group exhibited significantly higher retention rates for all three vitamins compared to all other groups. This indicates that the 4:4:2 combination ratio maintains the most comprehensive and stable antioxidant efficacy during prolonged storage. Consequently, the 4:4:2 formulation of EGCG: TP: dl-α-Toc represents the optimal combination for protecting multiple fat-soluble vitamins in this system (comparative HPLC analysis of samples is shown in the Supplementary File Figure S10).

### 3.5. Shelf-Life Prediction with Different Ratios of Natural Antioxidants

This study predicted the theoretical shelf life at 25 °C for various antioxidant blend samples based on the degradation of heat-sensitive vitamins during accelerated storage. The predicted shelf-life values were ranked as follows: 4:4:2 > 3:5:2 > 2:6:2 > 5:3:2 > 6:2:2. Key findings indicate that the 4:4:2 ratio (EGCG: TP: dl-α-Toc) composite system exhibits the longest theoretical shelf-life (exceeding 84 days), retaining 83% of the initial vitamin A concentration. This concentration is still higher than the 80% regulatory threshold specified in GB 28050-2011, and it exhibits significantly superior protective effects compared with other ratio combinations, while all other groups were lower than this value. The vitamin A retention rates were 78% for the 3:5:2 ratio, 76% for the 2:6:2 ratio, 69% for the 5:3:2 ratio, and 68% for the 6:2:2 ratio, respectively. All other groups exhibited retention values below 80% by day 21, while the 4:4:2 formulation maintained significantly superior protective effects (*p* < 0.05). Consequently, the predicted theoretical shelf-life at 25 °C for the 4:4:2 formulation exceeds 84 days, whereas all other formulations have predicted shelf-lives below this value. This result reveals that within the complex system of protein beverages, the molecular structural differences of antioxidants, their hydrophilic-lipophilic distribution, and the intermolecular interactions between them collectively determine the ultimate strength of the synergistic effect [34].

Existing research [35] indicates that the optimal example (4:4:2) likely represents a highly efficient synergistic network of redox cycle regeneration and multiphase coverage. TP and EGCG, as potent hydrogen donors, rapidly quench aqueous and interfacial radicals, whilst dl-α-Toc acts as an excellent lipid-phase radical chain reaction terminator. Furthermore, EGCG (highly hydrophilic), TP (moderately hydrophilic/interfacially active), and dl-α-Toc (highly lipophilic) achieve comprehensive distribution within the emulsion system, suggesting that the 4:4:2 ratio achieves an optimal balance in antioxidant distribution across the oil-water interface. Moreover, when multiple phenolic compounds coexist, they do not invariably exhibit synergy. High concentrations of TP or EGCG may competitively bind to proteins within the system through self-polymerisation or oxidation. Imbalanced ratios (e.g., excessively high EGCG) may induce antagonistic effects, increasing susceptibility to ineffective degradation [11].

Therefore, the ranking of predicted shelf-life values provides compelling evidence that combinatorial effects within a composite system comprising structurally similar yet polarity-diverse polyphenols and classical fat-soluble antioxidants are optimal. The 4:4:2 ratio identified in this study represents the optimal solution achieved within a specific protein matrix, balancing multiple factors including multi-mechanism coverage, intermolecular interactions, and protein-polyphenol complexation. This discovery not only provides direct guidance for developing high-performance antioxidant formulations for protein beverages but also deepens our understanding of the combined effects of multi-component antioxidants within complex food systems.

## 4. Conclusions

This study optimized an emulsion system, identifying 0.35% microcrystalline cellulose (MCC) as the optimal stabilizer. While the in vitro antioxidant activity ranked as vitamin C > TP > EGCG > PQQ > dl-α-Toc, vitamin protection in the actual emulsion followed the order of TP > EGCG > vitamin C > PQQ > dl-α-Toc, highlighting the critical role of molecular stability within complex matrices. Notably, a 4:4:2 mass ratio blend of EGCG, tea polyphenols (TP), and dl-α-Toc was identified as the optimal antioxidant combination, providing the most comprehensive and sustained protection for vitamins A, D_2_, and D_3_ over 21 days. The findings demonstrate that synergistic, two-phase antioxidant networks—specifically between fat-soluble dl-α-tocopherol and water-soluble polyphenols (TP and EGCG)—are more significant than in vitro radical scavenging capacity alone. Practically, this research offers a ready-to-use formulation strategy: using MCC as a stabilizer alongside this specific 4:4:2 antioxidant blend to significantly enhance the nutritional retention of sensitive fat-soluble vitamins in functional beverages.

This work provides a synergistic solution to the challenges of physical instability, chemical deterioration, and nutrient loss in protein beverages, offering a robust theoretical and technical framework for developing high-quality, nutritionally stable products. Despite these promising findings, the conclusions are currently restricted to the specific protein-based matrix tested, and further validation in other beverage types (e.g., fruit juices, plant-based milks) is needed. Additionally, industrial-scale production and real-time storage studies are required to confirm scalability and practical applicability. Future work will focus on extending this antioxidant strategy to other emulsion systems and optimizing the formulation for commercial production. Furthermore, intermediate temperature validation (e.g., at 35 °C) was not performed, which is a limitation of this study. Future work should include validation at intermediate temperatures to confirm the accuracy of the shelf-life prediction.

## Figures and Tables

**Table 1:** The effect of adding different natural antioxidants to the system on the retention of vitamins during storage acceleration.

Group	Time								
	0W			1W			2W		
	A	D_2_	D_3_	A	D_2_	D_3_	A	D_2_	D_3_
EGCG	33.38 ± 0.6 ^Aa^	1.45 ± 0.02 ^Aa^	1.43 ± 0.01 ^Aa^	15.3 ± 0.42 ^Ab^	0.86 ± 0.06 ^Ba^	0.81 ± 0.01 ^Ba^	7.1 ± 0.62 ^Aab^	0.25 ± 0.02 ^Cb^	ND
PQQ	26.4 ± 0.32 ^Aa^	1.09 ± 0.02 ^Ac^	1.04 ± 0.07 ^Ab^	11.56 ± 0.4 ^Bb^	0.68 ± 0.01 ^Ca^	0.76 ± 0.02 ^Bb^	<LOQ	ND	ND
TP	32.64 ± 0.82 ^Aab^	1.37 ± 0.02 ^Aa^	1.38 ± 0.02 ^Aab^	16.64 ± 0.42 ^Bab^	0.77 ± 0.01 ^Ba^	0.79 ± 0.01 ^Bab^	9.09 ± 0.24 ^Ca^	0.32 ± 0.01 ^Ca^	<LOQ
Vitamin C	29.62 ± 0.14 ^Ab^	1.24 ± 0.03 ^Ab^	1.24 ± 0.04 ^Ab^	18.87 ± 0.14 ^Ba^	0.79 ± 0.01 ^Ca^	0.51 ± 0.02 ^Bc^	6.0 ± 0.2 ^Bb^	ND	ND
dl-α-Toc	27.59 ± 0.19 ^Aa^	1.13 ± 0.04 ^Ab^	1.1 ± 0.05 ^Ab^	9.48 ± 0.14 ^Bc^	0.72 ± 0.01 ^Ba^	0.55 ± 0.01 ^Bc^	ND	ND	ND
control	8.34 ± 0.36 ^c^	0.67 ± 0.06 ^d^	0.58 ± 0.03 ^d^	ND	ND	ND	ND	ND	ND

Note. Different uppercase letters indicate significant differences between samples taken at different times within the same sample group. Different lowercase letters indicate significant differences between samples taken at the same time within the same sample group. *p* < 0.05. ND: Not detected.

**Table 2:** The effect of adding different ratios of EGCG: TP: dl-α-tocopherol to the system on accelerating vitamin retention during storage.

Group (EGCG:TP:dl-α-Toc)	Time											
	0W			1W			2W			3W		
	A	D_2_	D_3_	A	D_2_	D_3_	A	D_2_	D_3_	A	D_2_	D_3_
6:2:2	37.33 ± 0.27 ^Aa^	1.44 ± 0.03 ^Ab^	1.513 ± 0.01 ^Ab^	35.25 ± 0.16 ^Be^	1.39 ± 0.01 ^Ac^	1.5 ± 0.01 ^Ac^	32.3 ± 0.12 ^Ce^	1.34 ± 0.01 ^Ac^	1.45 ± 0.04 ^Ad^	30.89 ± 0.29 ^Cf^	1.32 ± 0.02 ^Ad^	1.42 ± 0.05 ^Ad^
5:3:2	39.13 ± 0.11 ^Aa^	1.51 ± 0.02 ^Ab^	1.52 ± 0.01 ^Ab^	36.43 ± 0.13 ^Ad^	1.49 ± 0.02 ^Ab^	1.51 ± 0.01 ^Ab^	33.6 ± 0.14 ^Ad^	1.48 ± 0.02 ^Bb^	1.49 ± 0.01 ^Bc^	31.21 ± 0.15 ^Be^	1.34 ± 0.05 ^Bd^	1.45 ± 0.03 ^Bc^
4:4:2	43.23 ± 0.61 ^Aa^	1.93 ± 0.02 ^Aa^	1.96 ± 0.01 ^Aa^	40.21 ± 0.35 ^Aa^	1.92 ± 0.01 ^Aa^	1.94 ± 0.01 ^Aa^	38.34 ± 0.24 ^Aa^	1.88 ± 0.02 ^Aa^	1.90 ± 0.01 ^Aa^	38.12 ± 0.33 ^Aa^	1.83 ± 0.01 ^Ba^	1.84 ± 0.02 ^Ba^
3:5:2	40.83 ± 0.35 ^Aa^	1.94 ± 0.01 ^Aa^	1.94 ± 0.01 ^Aa^	38.33 ± 0.26 ^Aa^	1.91 ± 0.01 ^Aa^	1.93 ± 0.01 ^Aa^	37.57 ± 0.14 ^Aa^	1.87 ± 0.03 ^Ab^	1.89 ± 0.02 ^Ab^	35.21 ± 0.1 ^Ab^	1.81 ± 0.02 ^Bb^	1.82 ± 0.02 ^Bb^
2:6:2	39.21 ± 0.18 ^Ab^	1.54 ± 0.02 ^Ab^	1.55 ± 0.02 ^Aa^	38.35 ± 0.11 ^Ab^	1.50 ± 0.01 ^Ab^	1.51 ± 0.01 ^Ab^	37.14 ± 0.12 ^Ab^	1.48 ± 0.01 ^Ab^	1.50 ± 0.01 ^Ac^	34.50 ± 0.40 ^Bc^	1.42 ± 0.01 ^Bc^	1.48 ± 0.01 ^Bc^

Note. Different uppercase letters indicate significant differences between samples taken at different times within the same sample group. Different lowercase letters indicate significant differences between samples taken at the same time within the same sample group. *p* < 0.05.

## Data Availability

The original contributions presented in this study are included in the article/Supplementary Materials. Further inquiries can be directed to the corresponding authors.

## Supplementary Materials

The following supporting information can be downloaded at https://www.mdpi.com/article/10.3390/foods15081392/s1. Figures S1–S3: Changes in ΔBS over storage time following addition of Na-CMC (Figure S1), Carrageenan (Figure S2), and MCC (Figure S3) to the system: (**a**) At 0 h; (**b**) Week 1; (**c**) Week 2; (**d**) Week 3. Figures S4–S7: Changes in ΔBS over storage time following addition of 0.15% (Figure S4), 0.25% (Figure S5), 0.35% (Figure S6), and 0.45% (Figure S7) MCC to the system: (**a**) At 0 h; (**b**) Week 1; (**c**) Week 2; (**d**) Week 3. Figure S8: Liquid chromatography graph of vitamin A (**a**), vitamin D_2_, and vitamin D_3_ (**b**). Figure S9: Comparative HPLC analysis of samples supplemented with EGCG (**a**), PQQ (**b**), TP (**c**), vitamin C (**d**), dl-α-Toc (**e**), and the control group (**f**). Figure S10: Comparative HPLC analysis of samples supplemented with EGCG:TP: dl-α-Toc = 6:2:2 (**a**), 5:3:2 (**b**), 4:4:2 (**c**), 3:5:2 (**d**), and 2:6:2 (**e**). Table S1: Standard calibration curve, LOD, LOQ, and correlation factors for each analyte.
