# Supplementary material for: Enhancing Stability of Vitamin-Fortified Protein Beverages: Optimization of Stabilizer Type and Concentration and Screening of Natural Antioxidant Combinations
Source: Foods. 2026 Apr 16;15(8):1392. doi: 10.3390/foods15081392 (PMC13115436; doi:10.3390/foods15081392)
Supplement: Supplementary file 1 [file foods-15-01392-s001.zip › foods-4236470-supplementary.pdf]

Supplementary Materials

Enhancing Stability of Vitamin-Fortified Protein Beverages: Optimization of Emulsifier Type and Concentration and Screening of Natural Antioxidant Combinations

Jiaxin Li [1], Sumei Ru [1], Linru Zhu [1], Yingshuang Lu [2], Junping Wang [1], Yan Zhang [2], Lu Dong [1,*] and Shuo Wang [2,*]

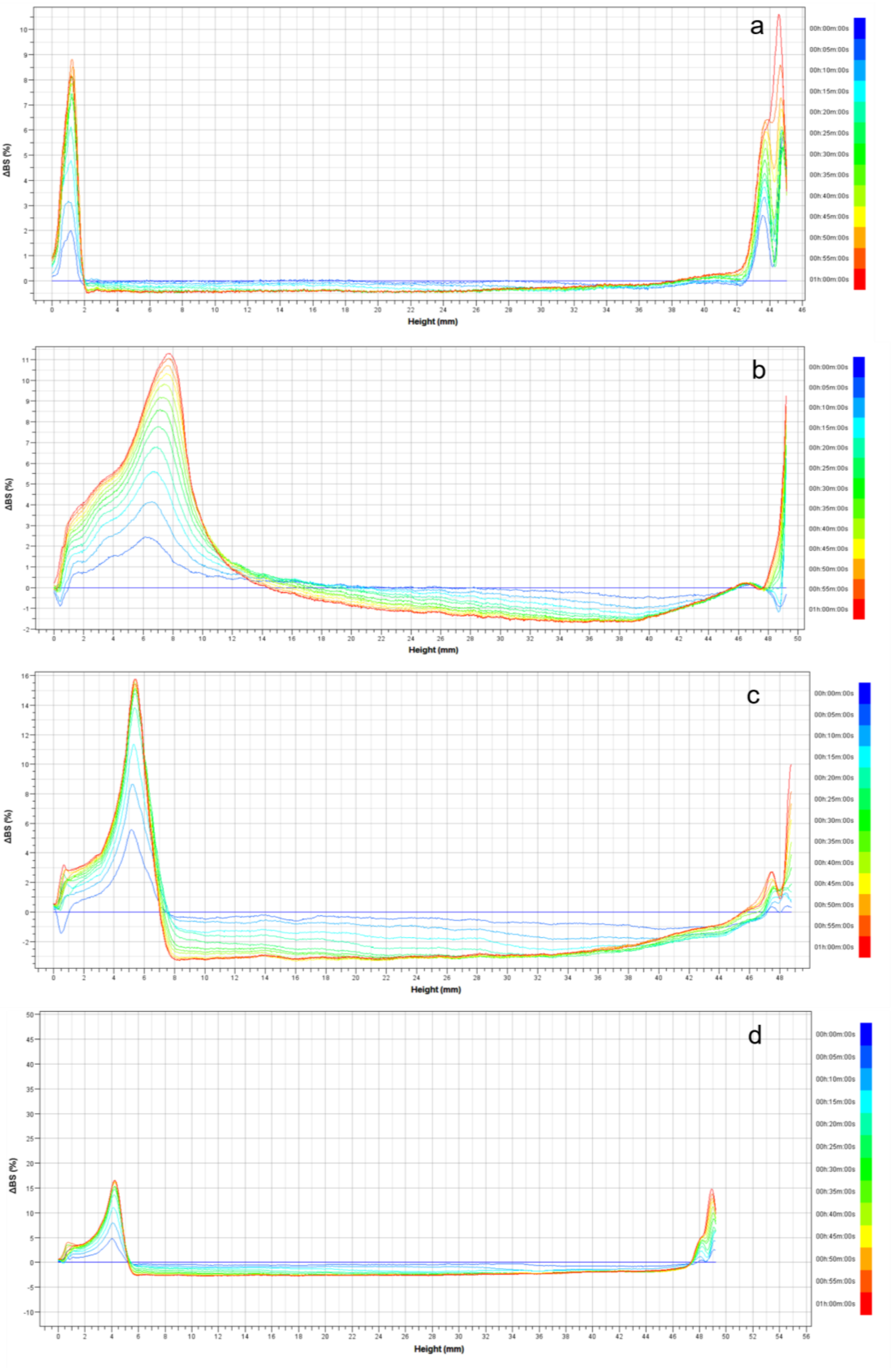

Figure S1. Changes in △BS over storage time following addition of Na-CMC to the system: (**a**) At 0 hours; (**b**) Week 1; (**c**) Week 2; (**d**) Week 3.

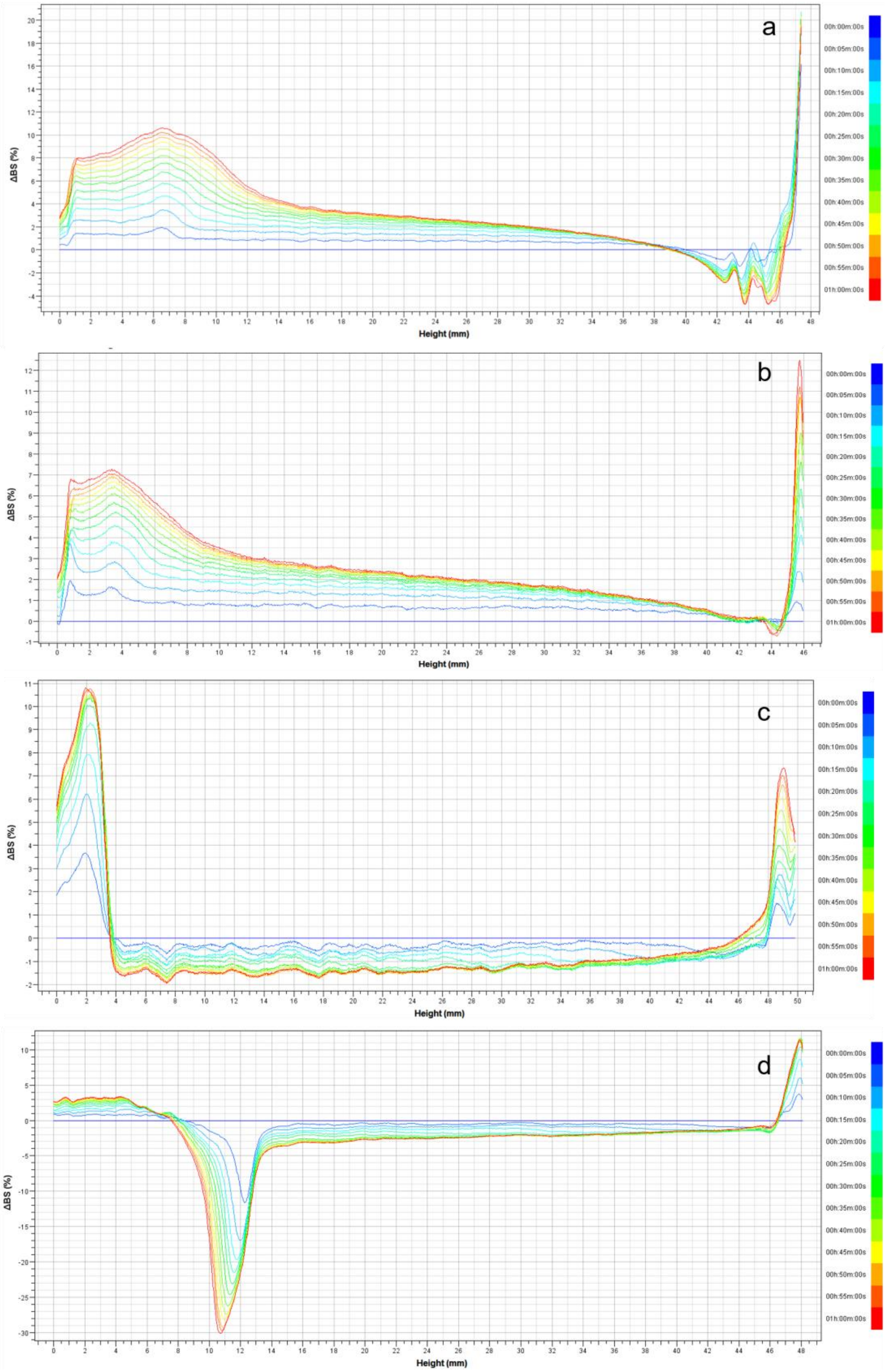

Figure S2. Changes in △BS over storage time following addition of Carrageenan to the system: (**a**) At 0 hours; (**b**) Week 1; (**c**) Week 2; (**d**) Week 3.

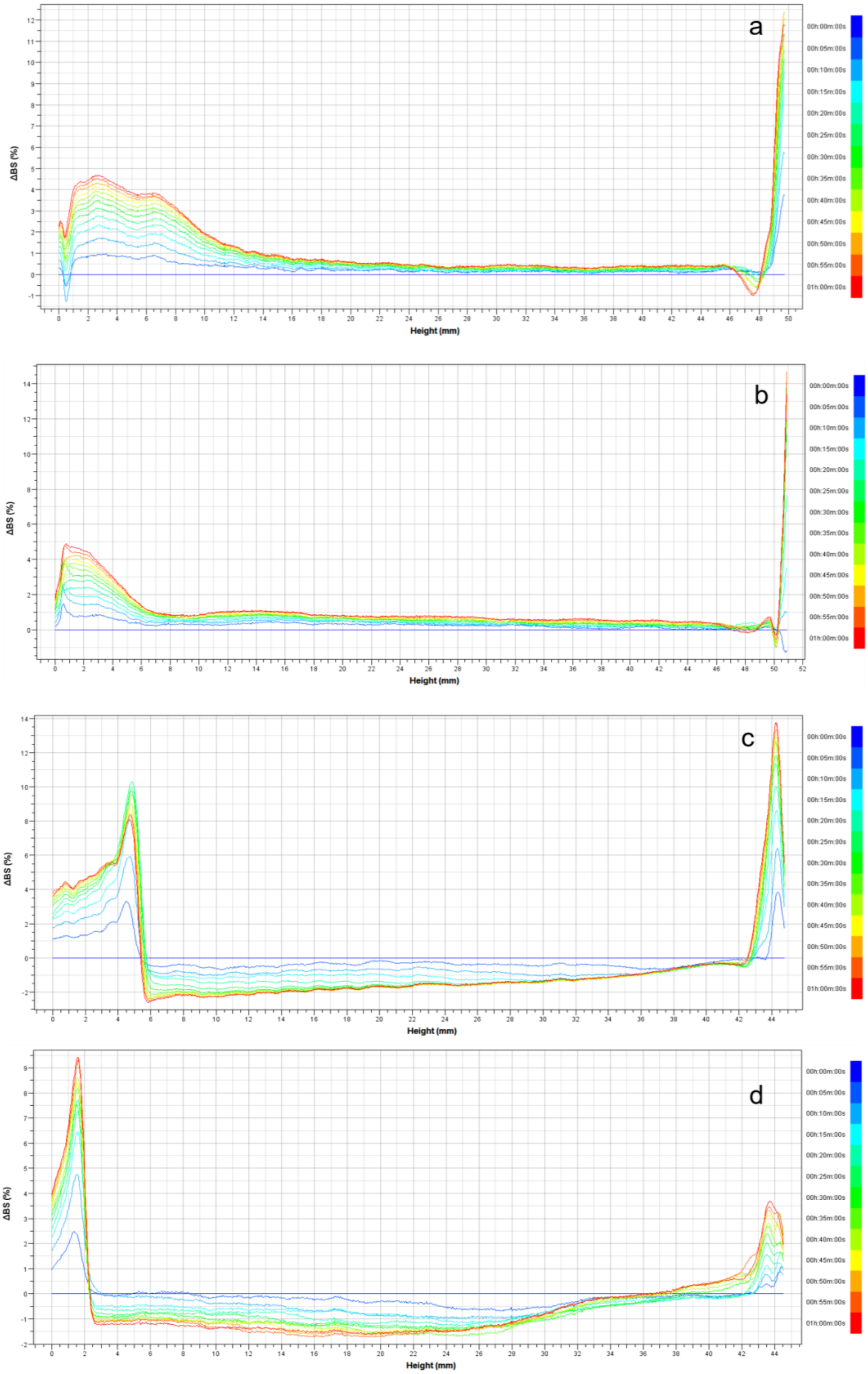

Figure S3. Changes in △BS over storage time following addition of MCC to the system: (**a**) At 0 hours; (**b**) Week 1; (**c**) Week 2; (**d**) Week 3.

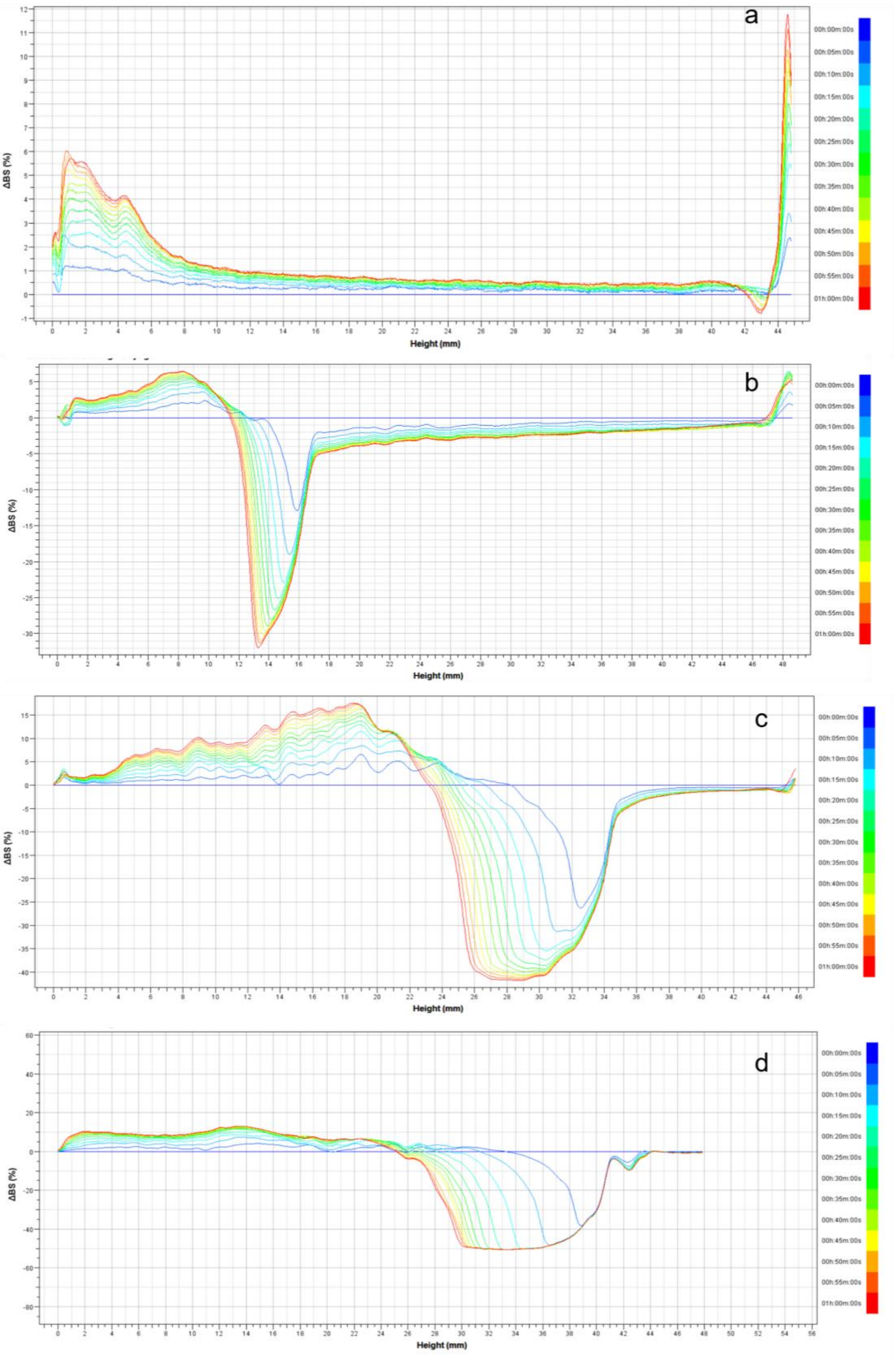

Figure S4. Changes in △BS over storage time following addition of 0.15% to the system: (**a**) At 0 hours; (**b**) Week 1; (**c**) Week 2; (**d**) Week 3.

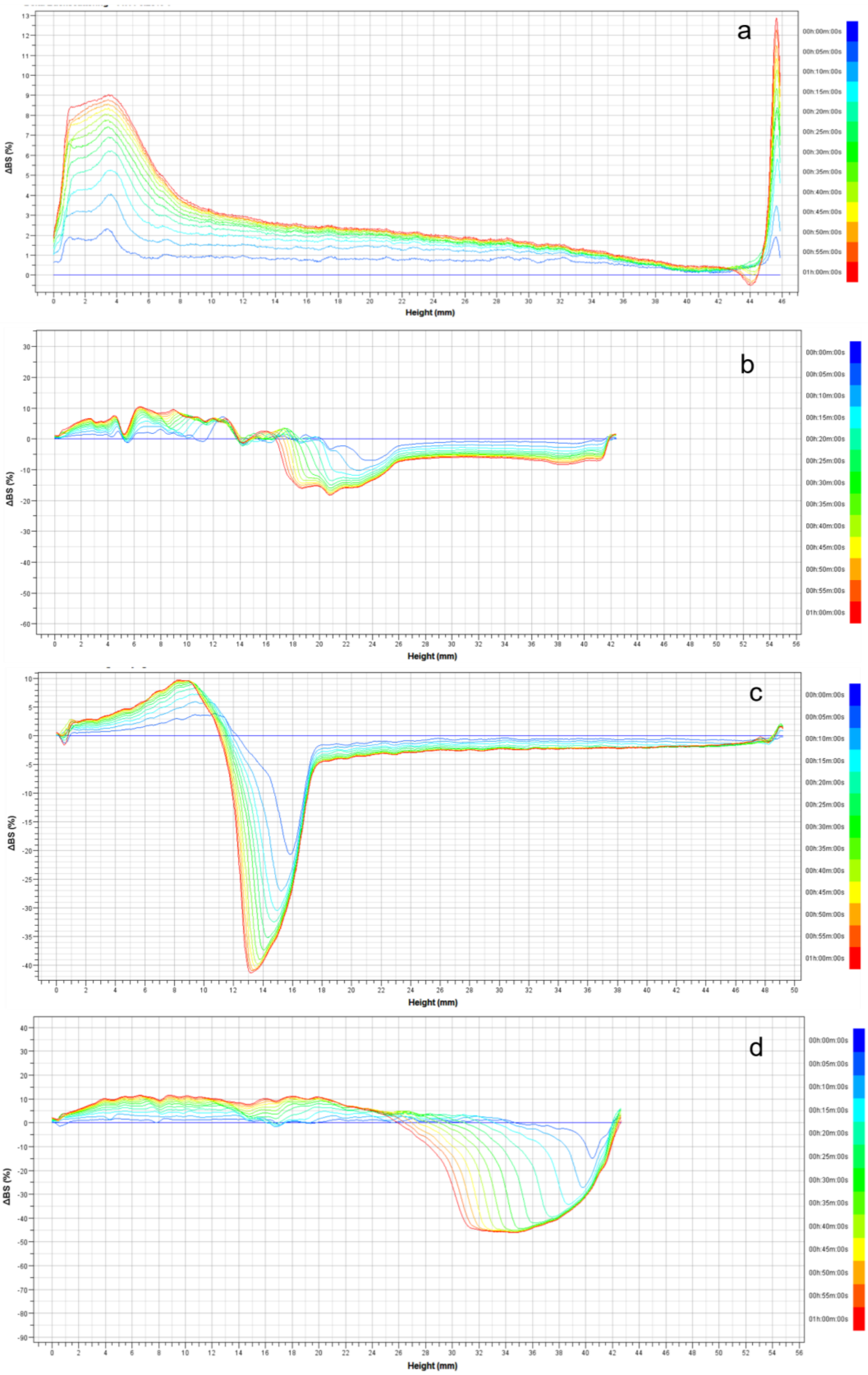

Figure S5. Changes in △BS over storage time following addition of 0.25% MCC to the system: (**a**) at 0 hours; (**b**) at week 1; (**c**) at week 2; (**d**) at week 3.

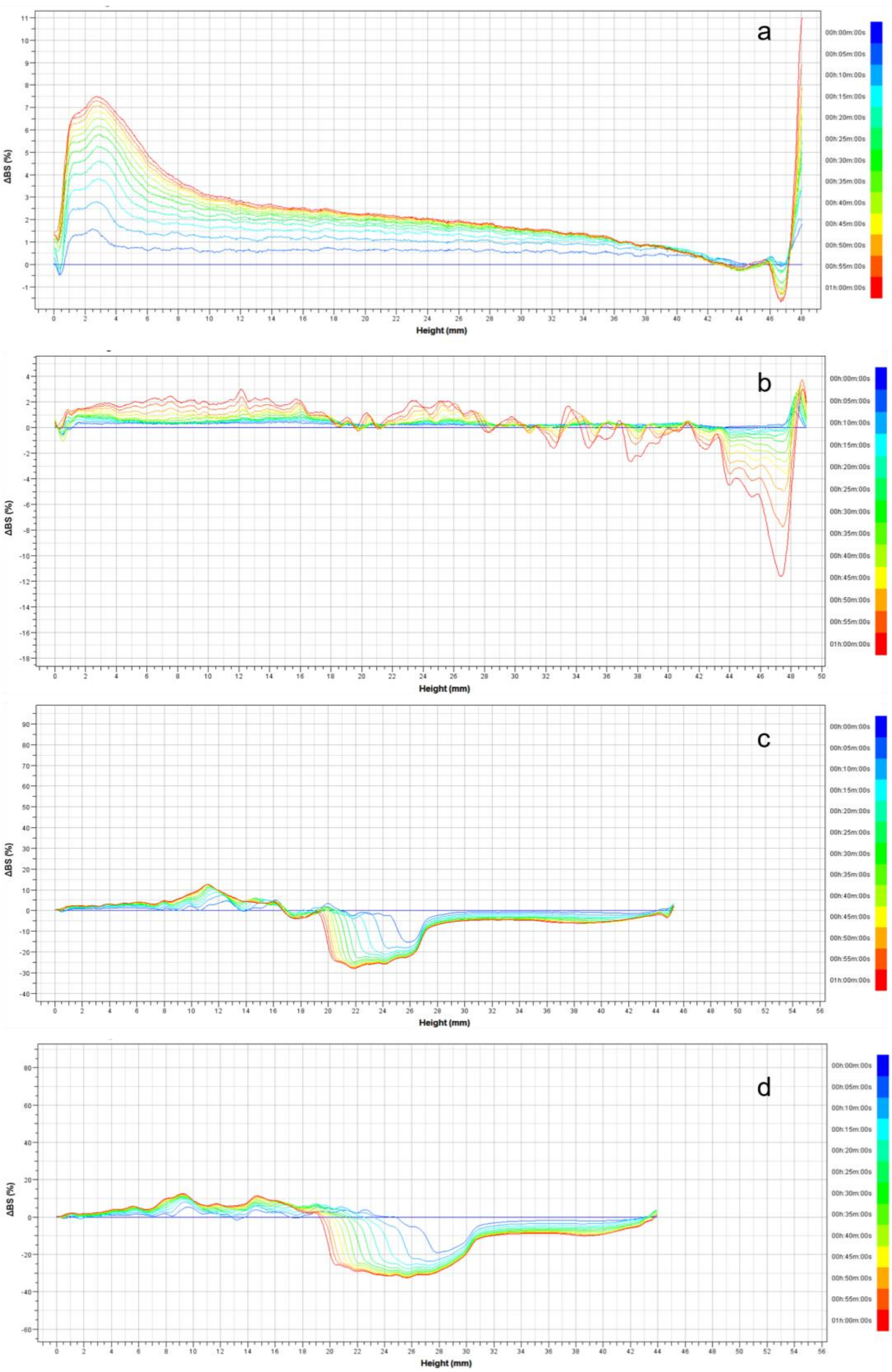

Figure S6. Changes in △BS over storage time following addition of 0.35% MCC to the system: (**a**) At 0 hours; (**b**) Week 1; (**c**) Week 2; (**d**) Week 3.

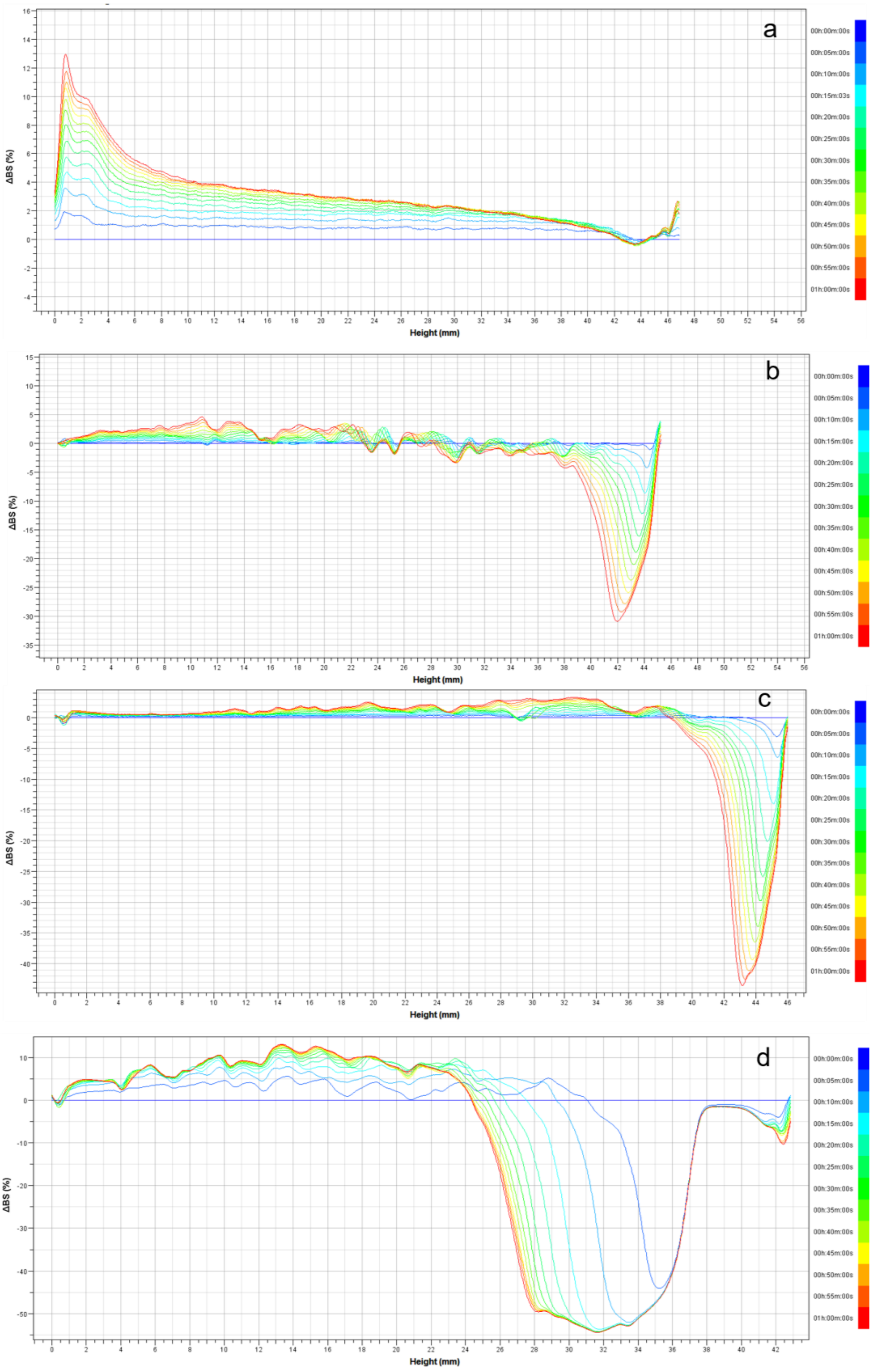

Figure S7. Changes in △BS over storage time following addition of 0.45% MCC to the system: (**a**) At 0 hours; (**b**) Week 1; (**c**) Week 2; (**d**) Week 3.

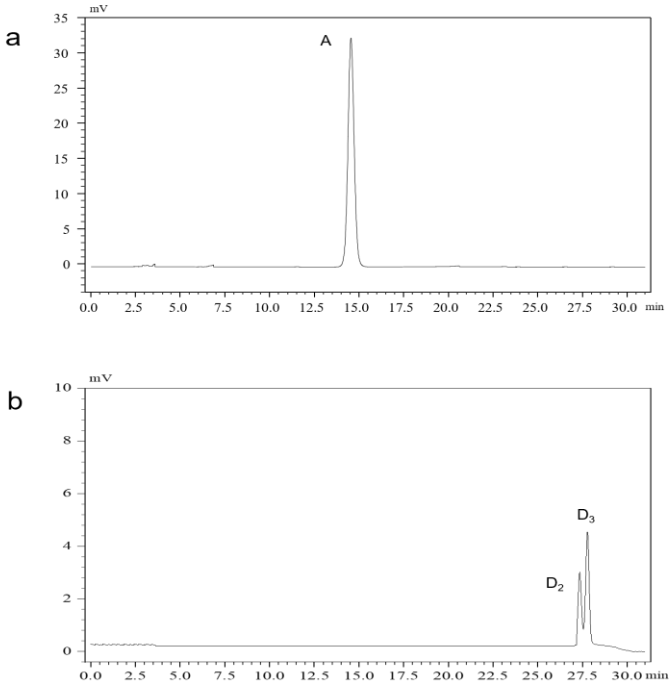

Figure S8. Liquid chromatography graph of vitamin A (**a**), vitamin D_2, and vitamin D_3 (**b**).

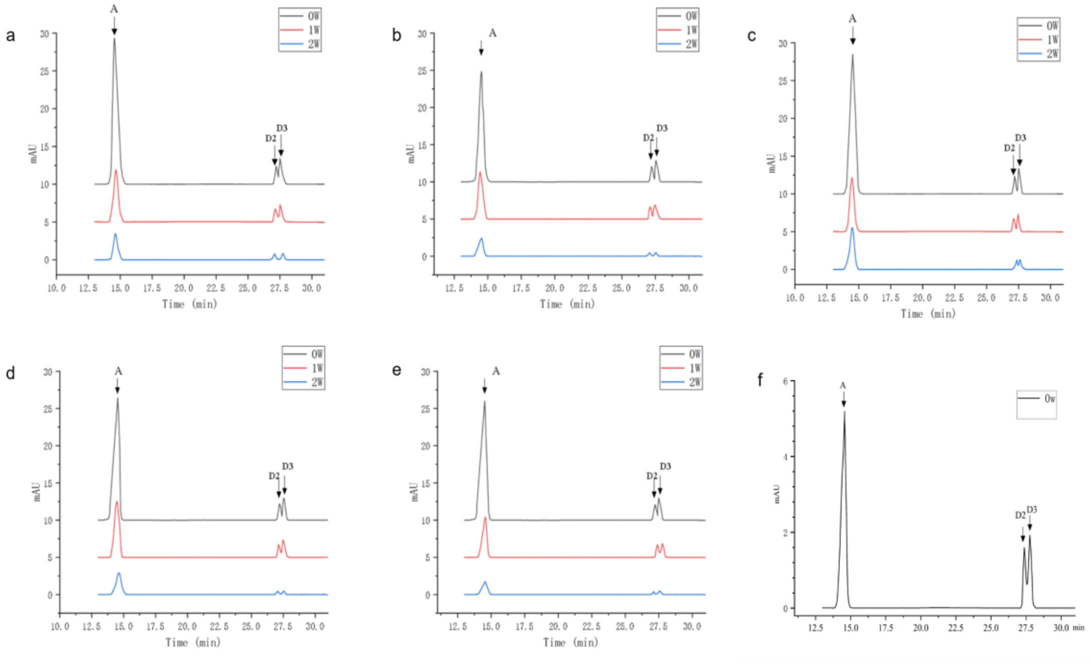

Figure S9. Comparative HPLC analysis of samples supplemented with EGCG (**a**), PQQ (**b**), TP (**c**), vitamin C (**d**), dl-α-Toc (**e**), and the control group (**f**).

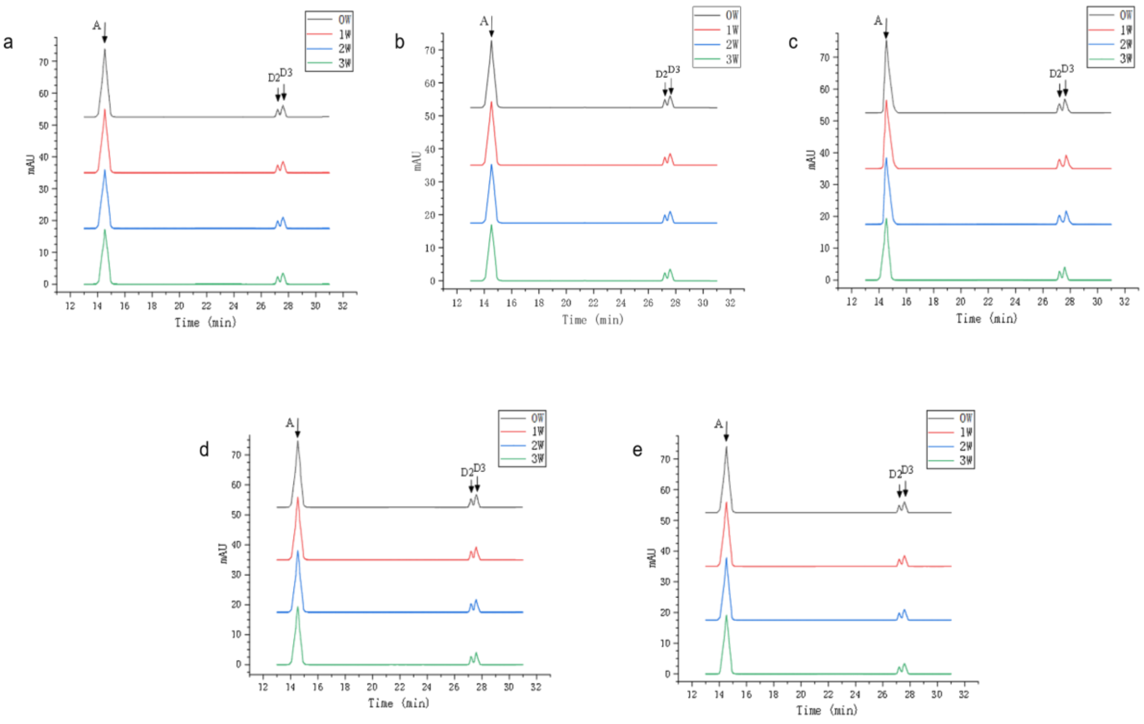

Figure S10. Comparative HPLC analysis of samples supplemented with EGCG:TP: dl-α-Toc= 6:2:2 (**a**),5:3:2 (**b**),4:4:2 (**c**),3:5:2 (**d**), and 2:6:2 (**e**).

Table S1. Standard calibration curve, LOD, LOQ, and correlation factors for each analyte.

Vitamins	Regression equation	LOD（ng/ml）	LOQ（ng/ml）	R^2
Vitamin A	Y=256099X+19368	25	86	0.9996
Vitamin D_2	Y=279339X+12338	1.0	3.0	0.9996
Vitamin D_3	Y=467381X+13722	6.7	23	0.9997
